# Fatal Case of Viral Pneumonia Associated with Metapneumovirus Infection in a Patient with a Burdened Medical History

**DOI:** 10.3390/microorganisms13081790

**Published:** 2025-07-31

**Authors:** Parandzem Khachatryan, Naira Karalyan, Hasmik Petunts, Sona Hakobyan, Hranush Avagyan, Zarine Ter-Pogossyan, Zaven Karalyan

**Affiliations:** 1 Department of Pathology, Yerevan State Medical University After Mkhitar Heratsi, Yerevan 0025, Armenia; parik82@gmail.com; 2 Department of Pathological Anatomy and Clinical Pathology, Yerevan State Medical University After Mkhitar Heratsi, Yerevan 0025, Armenia; 3 Laboratory of Cell Biology and Virology, Institute of Molecular Biology of NAS RA, Yerevan 0014, Armenia; 777sona7@gmail.com (S.H.);; 4 Experimental Laboratory, Yerevan State Medical University After Mkhitar Heratsi, Yerevan 0025, Armenia; 5 “Nairi” Medical Center, 21 Paronyan Str., Yerevan 0015, Armenia

**Keywords:** human metapneumovirus (hMPV), acute viral pneumonia, lung pathology, viral inclusions

## Abstract

**Background:** Human metapneumovirus (hMPV) is a respiratory pathogen that causes illness ranging from mild upper respiratory tract infections to severe pneumonia, particularly in individuals with comorbidities. Fatal cases of hMPV-induced hemorrhagic pneumonia are rare and likely under-reported. Diagnosis is often delayed due to overlapping symptoms with other respiratory viruses and the rapid progression of the disease. **Case presentation:** We report the case of a 55-year-old man with a complex medical history, including liver cirrhosis and diabetes mellitus, who developed acute viral pneumonia. Initial symptoms appeared three days before a sudden clinical deterioration marked by shortness of breath, hemoptysis, and respiratory failure. A nasopharyngeal swab taken on the third day of illness tested positive for hMPV by qRT-PCR. The patient died the following day. Postmortem molecular testing confirmed hMPV in lung tissue and alveolar contents. Autopsy revealed bilateral hemorrhagic pneumonia with regional lymphadenopathy. Histopathological examination showed alveolar hemorrhage, multinucleated cells, neutrophilic infiltration, activated autophagy in macrophages, and numerous cytoplasmic eosinophilic viral inclusions. **Conclusions:** This is the first documented case of fatal hMPV pneumonia in Armenia. It highlights the potential severity of hMPV in adults with chronic health conditions and emphasizes the need for timely molecular diagnostics. Postmortem identification of characteristic viral inclusions may serve as a cost-effective histopathological marker of hMPV-associated lung pathology.

## 1. Background

Human metapneumovirus (hMPV) belongs to the family Pneumoviridae, genus *Metapneumovirus*, and represents a negative-sense, single-stranded RNA virus. Although the virus was described relatively recently, in 2001 [1] it soon became clear that it was widespread, and humans can be easily infected by airborne droplets through direct contact with infected people. Now hMPV infection has been reported all around the globe [2]. Lethal hMPV infections in adults are usually observed in immunosuppressed patients [3]. In such cases, pneumonia most often develops [4]. In this case, report, we have studied postmortem pathological changes of the lungs (rapidly progressing pneumonia) in a non-elderly patient with a burdened anamnesis with several comorbidities, such as cirrhosis, diabetes mellitus, etc.

### 1.1. Case Presentation

The case under consideration in this report is a 55-year-old male with a complicated anamnesis.

### 1.2. Clinical History

The patient was hospitalized, complaining of shortness of breath and swelling in the testicular and paratesticular area. Concomitant diseases include liver cirrhosis, diabetes mellitus for 4–5 years, rib resection, and lung abscess drainage (5 years ago).

**Figure 1:**
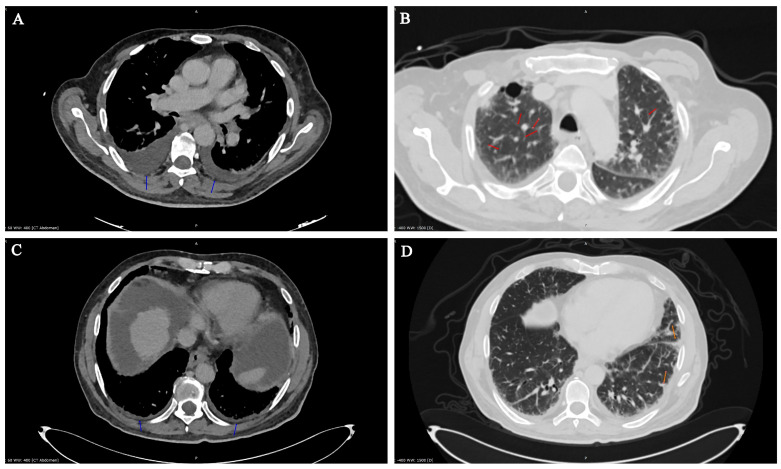
CT images obtained from patient in disease dynamics. CT scans from 25 November 2024 (**A**,**B**) and 7 January 2025 (**C**,**D**). Moderate (**A**) and minimal (**C**) pleural effusion on both sides (blue arrow): Diffuse interlobular septal thickening in the lungs (**B**) (red arrows). Localized ground-glass lesions, minimal consolidation zones (**D**) (orange arrows).

At admission on 24 November, consciousness was clear, the skin and visible mucous membranes were yellowish, and the limbs were slightly swollen with complaints of a slight fever, headache, and muscle aches. Symptoms like cough, sore or dry throat, nasal congestion, and nausea were not manifested. White blood cell count was 20.53 × 109/L, red blood cells 3.27 × 1012/L, and C-reactive protein was 115.34 mg/L (peak value). (Figure S1; Table S1) Total bilirubin 102.5 μmol/L, direct bilirubin 72.7 μmol/L, creatinine- 137 μmol/L, Gamma-glutamyl transferase 161.5 U/L, total protein 61.1 g/L, albumin 23.25 g/L, prothrombin time 34.8′′, international normalized ratio 2.73. An X-ray revealed subsegmental bilateral infiltration. CT scan showed moderate pleural effusion on both sides, diffuse interlobular septal thickening, and a postoperative cavity in the upper lobe of the right lung (Figure 1A,B). No lymphadenopathy was detected․ Treatment with antibiotics was started. Treatment with Azithromycin 500 mg once a day was started because of persistent fever for 5 days. Additionally, hepatoprotectors and gastroprotectors, antidiuretics, and infusion therapy and were prescribed. Normal Saline and 20% Albumine were used with Spirinolactone as an antidiuretic for infusion therapy to manage the edema. Omeprazole and Sucralfate were added for gastroprotection. Essentiale forte was the hepatoprotective drug. The dosages were strictly controlled, and laboratory parameters were strongly monitored (especially Na^+^, K^+^, Mg^2+^, glucose, creatinine, etc.).

Due to the worsening of the situation with increasing temperature, surgical intervention was performed on 13 December: orchiectomy from the right side with excision of Fournier’s gangrene.

The patient recovered smoothly after surgery, with some positive dynamic changes in the laboratory parameters, but the shortness of breath and weakness remained. On 7 January, the patient was urgently transferred to the intensive care unit with critical shortness of breath, cough, and hemoptysis.

CT observation less than 4 days before death revealed minimal pleural fluid on both sides (blue arrows) up to 0.8 cm thick (insignificant); also, on both sides, mostly on the left in the basal sections, minimally consolidated zones were observed (orange arrows) (Figure 1C,D). No pneumothorax was observed; increased thickening of interlobular septa in the lungs was observed, and postoperative cavity in the upper lobe of the right lung was 3.8 cm. The hardened zone in the pleural region of the 2nd–3rd intercostal space on the right 3.3 × 3.0 cm—probable postoperative—was without dynamics. No lymphadenopathy was detected. The lumens of the trachea and main bronchi were free.

Saturation progressively decreased during the next three days despite treatment, and wet crackles were auscultated. Due to the severe condition of the patient, additional CT scanning for the last four days was impossible.

On 11 January, the patient died. In fact, the patient developed an acute form of viral pneumonia with typical symptoms of fever, cough, runny nose, chest pain, and headache. It is necessary to note the relative speed of pneumonia development: from the full development of symptoms to death, less than 96 h passed.

The autopsy revealed bilateral hemorrhagic pneumonia with lymphadenopathy. Lungs were totally heavy and congested, with subpleural hemorrhages, hemorrhagic and edematous tracheal and bronchial mucosa, and diffuse hemorrhagic consolidations on the cut surface.

### 1.3. Laboratory Testing

Laboratory testing was negative for autoimmune disease, including normal IgG and IgM cardiolipin antibodies, normal Beta-2-Glycoprotein IgG and IgM antibodies, negative antinuclear antibodies, negative antineutrophil cytoplasmic antibodies, negative rheumatoid factor, and negative antiglomerular basement membrane antibody. Infection diagnostic tests included negative HIV-1/2 antibodies and HIV-1 antigen, negative Legionella and pneumococcal antigens, negative lung Herpesviridae (Herpes simplex virus 1; Herpes simplex virus 2; Epstein–Barr, cytomegalovirus); negative SARS-CoV-2 (measured by quantitative reverse transcription polymerase chain reaction (qRT-PCR); negative hepatitis viral panel, negative Aspergillus galactomannan antigen and galactomannan index, negative mycoplasma, negative dengue virus presence (procedure related to imported cases of Dengue fever in tourists from Armenia, measured by qRT-PCR). The influenza virus was excluded while the patient was still alive in the clinic, and its absence was confirmed postmortem.

Laboratory analysis confirmed the presence of hMPV by qRT-PCR [5]. The primers used for the detection of the human metapneumovirus (hMPV) N gene (Forward: 5′-GAGTCTCAGTACACAATAA-3′; Reverse: 5′-GCATTTCCGAGAACAACAC-3′) were adopted from the protocol described by Côté [6]. In this study, two SYBR green real-time RT-PCR assays (absolute and relative quantification) were used to quantitate hMPV. A diagnosis for respiratory syncytial virus was also performed with negative results.

**Figure 2:**
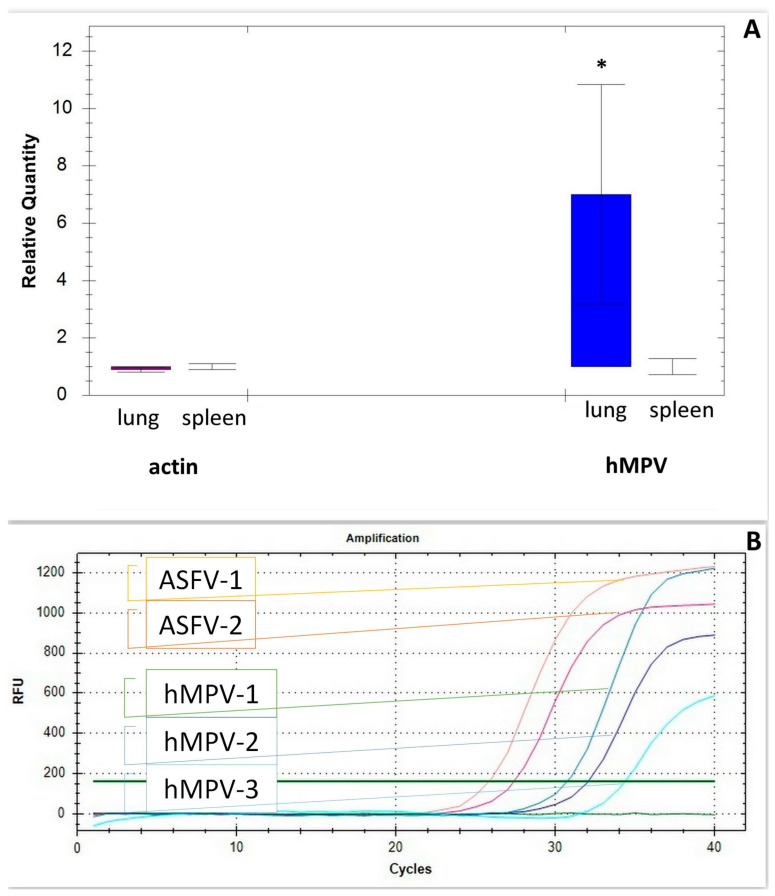
Real-time RT-PCR of hMPV. (**A**). Human metapneumovirus cDNA in human lung and spleen template run on the Bio-Rad CFX96™ Real-Time PCR Detection System * significant compared with all measurements (*p* < 0.05). (**B**). Amplification plot and the external standard curves generated by decimal logarithmic dilutions of African swine fever virus gene cDNA (shades of red) and nMPV cDNA (shades of blue). The levels of African swine fever virus cDNA (gene B646L) obtained in vitro [5] are compared with the levels of nMPV cDNA in lung tissue.

hMPV was detected after hospitalization on the third day by RT-PCR from a nasopharyngeal swab and postmortem on 11 January 2025 from lung samples. hMPV was detected by RT-PCR both in bronchoalveolar lavage and in lung tissue. Blood and internal organ samples were also tested for the presence of hMPV, the data were negative (absence of viremia). Figure 2 shows the data on the rtPCR of hMPV in the patient. Data on the relative content of viral cDNA in the lungs (significantly higher compared to all other measurements) and spleen are presented in comparison with actin cDNA levels (Figure 2A). Figure 2B shows decimal logarithmic dilutions of African swine fever virus gene cDNA (shades of red) and nMPV cDNA (shades of blue). The levels of African swine fever virus cDNA (gene B646L) obtained in vitro [5] are compared with the levels of nMPV cDNA in lung tissue.

### 1.4. Pathological Findings

**Figure 3:**
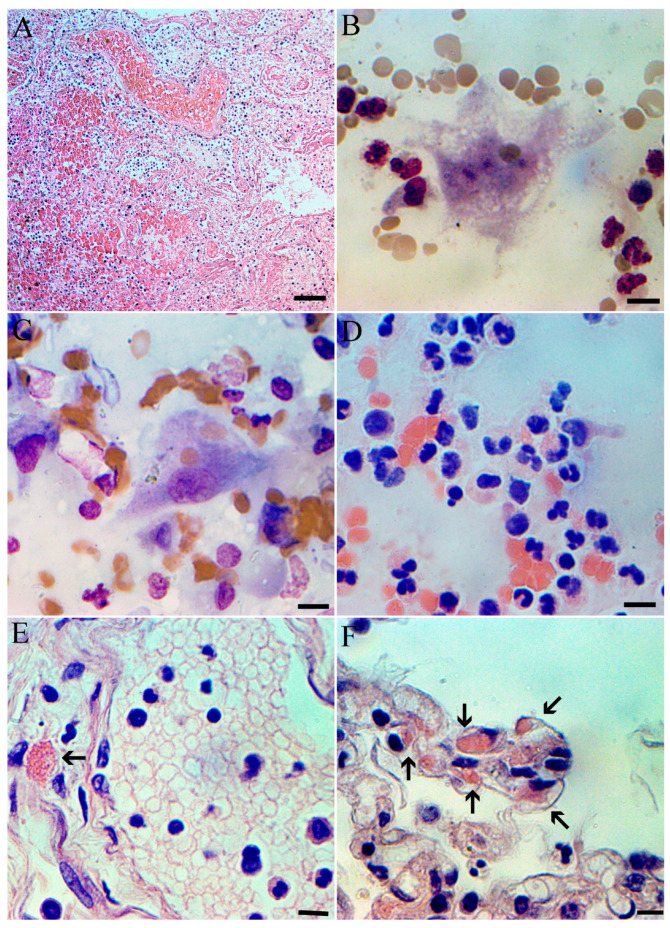
Postmortem histopathology and cytopathology of lung at hMPV. Lung with hemorrhages in intra- and interalveolar spaces, focal rupture of interalveolar septae and interstitial lymphocytic infiltration; H&E, scale bar 100 µm. (**A**). Multinuclear cell, probably alveolar macrophage from lung smears. Staining by Pappenheim; scale bar 10 µm. (**B**). Activated alveolar macrophage with phagocytized erythrocytes. Lung smears, staining by Pappenheim; scale bar 10 µm. (**C**). Lung smear with multiple neutrophils staining by H&E; scale bar 10 µm. (**D**). Section of lung with necrosis in the lumen of the vein and hMPV eosinophilic inclusion in cytoplasm (arrowed) scale bar 10 µm. (**E**). Section of lung with multiple hMPV eosinophilic inclusions in cytoplasm (arrowed) scale bar 10 µm. (**F**).

Postmortem examination of the lungs starts six hours after death. Cut sections from lungs stained with routine hematoxylin–eosin revealed edema, hemorrhages in intra- and interalveolar spaces, with focal rupture of interalveolar septae and interstitial lymphocytic infiltration (Figure 3A). Postmortem lung aspirate revealed large numbers of neutrophils, lymphocytes, erythrocytes and epithelial cells (predominantly airway mucosa).

Lung impression smears prepared routinely [7] and stained both with Pappenheim and with hematoxylin and eosin. Multinucleated cells, predominantly of macrophage origin, are easily identified on the impression smears (Figure 3B). A significant proportion of pulmonary macrophages show signs of autophagy (Figure 3C), which is an important part of pulmonary pathology [8]. Lung impression smears also revealed a high number of leukocytes, primarily neutrophils (Figure 3D). Histological studies revealed an eosinophilic inclusions in the cytoplasm of epithelial cells, which are characteristic of mononegaviral pathology, and in particular hMPV (Figure 3E,F). Compared to other mononegaviruses, eosinophilic inclusions found in alveolocytes are quite large, occur frequently and are easily detected.

## 2. Discussion

Diffuse alveolar hemorrhage is a severe, life-threatening lung condition that requires emergency medical care. This pathology can develop with viral lung lesions and, in particular, with metapneumovirus infection [9,10]. Typically, alveolar hemorrhage is characterized by the accumulation of red blood cells in the alveoli, from the bronchial vessels, pulmonary vessels, or microcirculation [10]. In our patient, the diagnosis was relatively similar to that described in [10], and the hemorrhage developed against the background of untreated end-stage liver disease, decompensated liver cirrhosis and, therefore, an underlying predisposition to bleeding.

Human metapneumovirus belongs to the order Mononegavirales is an enveloped, non-segmented, negative-sense, single-stranded RNA viruses. hMPV is very common, has been isolated on all continents and usually has a seasonal distribution, with outbreaks primarily occurring in the spring and winter months and accounting for up to 11% of respiratory tract infections [10]. Typically, hMPV causes upper and lower respiratory tract infections in young children, in adults this virus causes serious infections only in immunosuppressed patients [11,12]. The patient described in this article had an aggravated clinical history, but with relatively moderate immunosuppression. The case of rapidly developing bilateral hemorrhagic pneumonia described by us demonstrates the vulnerability of patients with an aggravated history to the development of a lethal form of metapneumovirus infection. In general, our data coincide with the clinical characteristics of the development of hMPV in adult patients [13,14,15]. The rapid increase in symptoms ending in a fatal outcome stands out somewhat.

The cytoplasmic inclusions induced by hMPV are somewhat similar to viral inclusions formed by other mononegaviruses, rabies and measles viruses, and probably represent an essential component of the life cycle of many negative-sense RNA viruses [16,17]. It can be theoretically assumed that viral cytoplasmic inclusions in immunocompromised individuals infected with hMPV, as in other mononegavirus infections, are usually larger in size, more numerous and better visible under light microscopy. This may be associated with a higher viral load, longer viral shedding, and therefore an increased risk of pneumonia and possible death.

qRT-PCR revealed relatively high levels of hMPV genome copies, the content of which in human lung tissue was slightly lower than those of the African swine fever virus gene transcripts (taken as a standard) in vitro (where viral transcript levels are usually higher).

This case underscores the importance of including hMPV in the differential diagnosis of acute viral pneumonia, particularly in patients with multiple comorbidities or atypical clinical presentations. The rapid progression of illness in this patient—from mild symptoms to death in under four days—highlights the aggressive nature of hMPV in vulnerable individuals, even outside classical high-risk groups such as the immunocompromised.

qRT-PCR diagnostics in clinical and postmortem settings proved essential for identifying hMPV as the etiological agent, especially when conventional bacterial and viral panels were negative. Moreover, the detection of characteristic eosinophilic cytoplasmic viral inclusions in lung tissue may serve as a reliable histopathological marker in cases where molecular testing is not immediately available or in resource-limited settings. The findings support incorporating molecular viral testing and targeted histopathology into the standard diagnostic workflow for rapidly progressing pneumonia cases, especially when initial treatments are ineffective and standard pathogens are not identified. Early recognition of hMPV could facilitate improved clinical decision-making, appropriate antiviral or supportive therapy, and public health tracking of severe respiratory infections.

## 3. Conclusions

We describe the first documented case of hMPV-associated death in Armenia. This case highlights the possible clinical impact of hMPV-associated pneumonia in older adults and the role of the virus in mortality. We would also like to point out the importance of integrating molecular diagnostics, in particular qRT-PCR assays, into diagnostics of lung diseases, especially in vulnerable populations. Such measures will improve early detection and thus patient outcomes. During a postmortem examination for final diagnosis, a fairly convenient and inexpensive method of confirming pulmonary pathology is the detection of characteristic viral inclusions (they are also observed with the respiratory syncytial virus) in the cytoplasm.

## Data Availability

The authors confirm that the data supporting the findings of this study are available within the article [and/or] its Supplementary Materials.

## Supplementary Materials

The following supporting information can be downloaded at: https://www.mdpi.com/article/10.3390/microorganisms13081790/s1, Figure S1: Timetable with dynamic changes of body temperature, saturation, and C-reactive protein; Table S1: Dynamic changes of complete blood count.

## Abbreviations

hMPV	human metapneumovirus
RT-PCR	reverse transcription polymerase chain reaction
qRT-PCR	quantitative reverse transcription polymerase chain reaction
IgG	immunoglobulin G
IgM	immunoglobulin M
SARS-CoV-2	severe acute respiratory syndrome coronavirus 2
CT	computed tomographyomputed Tomography
