# Supplementary material for: Fatal Case of Viral Pneumonia Associated with Metapneumovirus Infection in a Patient with a Burdened Medical History
Source: Microorganisms. 2025 Jul 31;13(8):1790. doi: 10.3390/microorganisms13081790 (PMC12388842; doi:10.3390/microorganisms13081790)
Supplement: Supplementary file 1 [file microorganisms-13-01790-s001.zip › Table 1S.pdf]

Table 1 S

	24/11	30/11	06/12	08/12	12/12	18/12	24/12	30/12	03/01	07/01	08/01	09/01	10/01	Reference Ranges
C-reactive protein	115.3	90.5	75.4	80.2	100.2	85.2	60.6	58.5	55.8	98.9	95.2	86.4	94.4	10mg/L
WBC	$20.53x10^9$	$18.63x10^9$	$15.54x10^9$	$10.24x10^9$	$18.21x10^9$	$12.74x10^9$	$10.36x10^9$	$11.57x10^9$	$9.55x10^9$	$21.10x10^9$	$18.58x10^9$	$16.10x10^9$	$15.98x10^9$	4.5-$11x10^9$ /L
RBC	3.27×10^{12}	3.8×10^{12}	3.3×10^{12}	4.0×10^{12}	3.87×10^{12}	3.7×10^{12}	3.25×10^{12}	4.0×10^{12}	3.8×10^{12}	3.7×10^{12}	3.2×10^{12}	3.3×10^{12}	3.27×10^{12}	4.5-$6x10^{12}$ /L
Total bilirubin	102.5	110.8	95.5	86.7	96.2	93.5	84.5	86.8	93.5	95.7	99.4	96.5	98.3	0 to 21 µmol/L
Direct bilirubin	72.7	72.7	64.0	54.4	52.4	52.7	45.8	42.1	63.0	72.7	61.3	58.7	62.0	0 to 5.1 µmol/L
Creatinine	137	146	152	121	156	147	189	184	178	236	254	235	257	53 to106 µmol/L
GGT	161.5	153.2	145.4	152.0	148.3	174.2	162.8	158.6	162.6	161.3	157.8	175.0	174.2	5-40U/L
Total protein	61.1	58.2	62.3	60.7	78.5	82.5	75.6	74.9	65.5	63.3	67.8	55.4	54.3	60-80g/L
Albumin	23.25	28.6	30.2	31.2	30.7	32.8	33.4	35.6	36.1	34.2	37.4	24.5	23.4	35-55g/L
PT	34.8’’	38.9’’	35.2’’	29.8’’	39.8’’	25.6’’	28.4’’	25.4’’	26.4’’	38.8’’	40.1’’	41.2’’	40.3’’	12-16’’

WBC – White Blood Cells; RBC – Red Blood Cells; GGT – Gamma Glutamyl Transferase; PT - Prothrombin Time,
