# Supplementary figures and images for: Fatal Case of Viral Pneumonia Associated with Metapneumovirus Infection in a Patient with a Burdened Medical History
Source: Microorganisms. 2025 Jul 31;13(8):1790. doi: 10.3390/microorganisms13081790 (PMC12388842; doi:10.3390/microorganisms13081790)

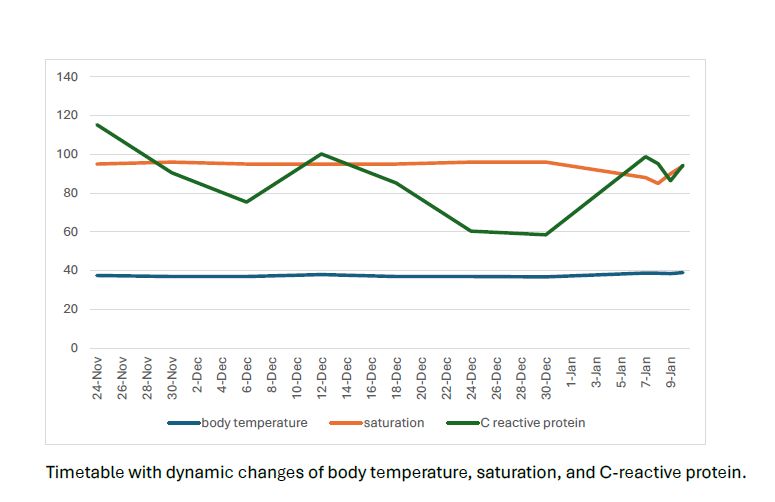

Supplement: Supplementary file 1 [file microorganisms-13-01790-s001.zip › Figure 1S.jpg]
